# Life-Course Regulation of Health and Disease by Nitric Oxide: Mechanistic Insights

**DOI:** 10.3390/antiox15040439

**Published:** 2026-04-01

**Authors:** Chien-Ning Hsu, You-Lin Tain

**Affiliations:** 1 Department of Pharmacy, Kaohsiung Municipal Ta-Tung Hospital, Kaohsiung 801, Taiwan; cnhsu@cgmh.org.tw; 2 Department of Pharmacy, Kaohsiung Chang Gung Memorial Hospital, Kaohsiung 833, Taiwan; 3 School of Pharmacy, Kaohsiung Medical University, Kaohsiung 807, Taiwan; 4 Department of Pediatrics, Kaohsiung Chang Gung Memorial Hospital, Kaohsiung 833, Taiwan; 5 Doctoral Program of Clinical and Experimental Medicine, National Sun Yat-Sen University, Kaohsiung 804, Taiwan; 6 College of Medicine, Chang Gung University, Taoyuan 333, Taiwan

**Keywords:** nitric oxide, nitric oxide synthase, asymmetric dimethylarginine, cardiovascular disease, chronic kidney disease, metabolic syndrome, developmental origins of health and disease (DOHaD)

## Abstract

Nitric oxide (NO) functions as a master integrative regulator of cardiovascular–kidney–metabolic (CKM) homeostasis, yet it displays a profound Janus face, defined by concentration- and context-dependent roles in both health and disease. This narrative review examines NO signaling from a life-course perspective, beginning with fetal programming, during which the NO–asymmetric dimethylarginine (ADMA) axis orchestrates placental development and nephron endowment. Perturbations during this critical window—such as maternal ADMA elevation—can imprint a maladaptive trajectory toward adult-onset hypertension and chronic kidney disease. In adulthood, this initially silent dysregulation of NO signaling is amplified by Western dietary patterns and environmental pollutants, culminating in the clinical manifestation of the CKM triad. This pathological transition is driven by eNOS uncoupling and ADMA accumulation, which shift redox balance toward peroxynitrite formation and precipitate mitochondrial bioenergetic failure. Moreover, while constitutive NO production is essential for vascular homeostasis, pathological induction of inducible NOS generates excessive NO fluxes that promote insulin resistance and tissue injury. With advancing age, a progressive loss of NO resilience further exacerbates multi-organ vulnerability. To mitigate the cumulative burden of CKM disease, this review highlights developmental reprogramming strategies—such as perinatal L-citrulline supplementation and ADMA-lowering interventions—as interventions to restore physiological NO signaling. Integrating such early-life strategies with contemporary pharmacological therapies offers a coherent framework for maintaining NO bioavailability and extending health span across the life course.

## 1. Introduction

Nitric oxide (NO) is a small, diffusible free radical signaling molecule that regulates vascular tone, cellular bioenergetics, immune responses, and redox homeostasis across multiple organ systems [1,2,3]. Rather than reiterating its well-established biochemical properties, this review briefly contextualizes canonical NO biology and focuses on its dynamic regulation across the life course. In this review, the “life-course” concept refers to the stage-dependent roles and regulation of NO signaling across early development, adulthood, and aging, reflecting differences in physiological requirements, environmental exposures, and susceptibility to disease. From fetal development to aging, NO bioavailability critically influences physiological resilience and disease susceptibility [4,5].

The biological impact of NO is determined by spatiotemporal context, concentration thresholds, and redox environment. Under physiological conditions, NO maintains endothelial integrity, optimizes tissue perfusion, and preserves metabolic flexibility [1,3]. Conversely, impaired synthesis or reduced bioavailability promotes oxidative and nitrosative stress, amplifying inflammatory signaling and tissue injury [2]. Across the life course, NO signaling fulfills distinct physiological roles. During early development, NO participates in organogenesis, vascular formation, and developmental programming. In adulthood, NO predominantly supports vascular homeostasis, metabolic regulation, and organ function. During aging, however, reduced NO bioavailability and increased oxidative stress contribute to endothelial dysfunction and the progression of cardiorenal and metabolic disorders. Within a life-course and Developmental Origins of Health and Disease (DOHaD) framework [6,7], early-life perturbations in NO signaling may durably program cardiovascular [8], renal [9], and metabolic vulnerability [10].

Cardiovascular disease (CVD), chronic kidney disease (CKD), and metabolic disorders remain major global health burdens [11,12]. Increasing evidence indicates that these conditions form an interconnected pathophysiological network, conceptualized as the cardiovascular–kidney–metabolic syndrome (CKMS) [13]. CKMS reflects coordinated dysfunction across heart, vasculature, kidney, liver, and metabolic tissues, with shared mechanistic substrates that include NO dysregulation. Epidemiological data indicate that a large proportion of adults exhibit at least one CKMS feature [14], underscoring the need for mechanistic models that integrate inter-organ crosstalk and early-life programming.

This review therefore repositions NO biology within a life-course framework, emphasizing developmental programming, redox-dependent modulation, and organ-system integration in CKMS, rather than re-examining established biochemical principles.

## 2. Materials and Methods

In view of the broad conceptual scope and substantial heterogeneity of the existing literature, a narrative review design was selected in preference to systematic or scoping methodologies, allowing for an integrative evaluation of evolving concepts across NO biology, developmental science, redox signaling, experimental models, and clinical medicine. A structured literature search was performed to capture relevant English-language publications issued between January 2000 and December 2025. Both preclinical and clinical studies were retrieved from the Cochrane Library, PubMed/MEDLINE, and Embase databases. The initial search yielded approximately 18,500 records (PubMed ≈ 12,300, Embase ≈ 6000, Cochrane ≈ 200), which were screened by title/abstract and full text, with 755 studies meeting inclusion criteria for narrative synthesis.

The search strategy employed combinations of terms related to NO and its metabolic and redox pathways (“nitric oxide”, “nitrite”, “nitrate”, “asymmetric dimethylarginine”, “symmetric dimethylarginine”, “arginine”, “oxidative stress”, “reactive oxygen species”, “reactive nitrogen species”, and “peroxynitrite”), developmental programming and life-course biology (“DOHaD”, “developmental programming”, “life-course theory”, “offspring”, “maternal”, “pregnancy”, “lactation”, and “reprogramming”), as well as CKMS phenotypes (“obesity”, “metabolic syndrome”, “cardiovascular disease”, “heart failure”, “hypertension”, “dyslipidemia”, “chronic kidney disease”, “insulin resistance”, “diabetes”, “atherosclerosis”, and “hepatic steatosis”). In addition, the reference lists of all eligible articles were manually examined to identify further relevant publications.

This integrative search and selection framework enabled a coherent synthesis of mechanistic and translational evidence addressing the role of NO in life-course regulation of cardiometabolic and kidney health and disease. During the preparation of this work, ChatGPT (version 5, OpenAI) was used for language editing to improve clarity and readability. NotebookLM (Google) assisted in figure conceptualization and layout planning. All final images were created from original sketches prepared by the authors, and no AI tool generated the final figures.

## 3. Physiological Roles of NO in Health

### 3.1. NO Generation

NO production arises from enzymatic and non-enzymatic pathways whose relative contributions vary across developmental stages and pathophysiological states [15]. Because these mechanisms have been extensively reviewed elsewhere, we provide only a concise overview here and refer readers to prior comprehensive reviews for detailed biochemical descriptions [3,15].

The nitric oxide synthase (NOS) family—endothelial (eNOS), neuronal (nNOS), and inducible (iNOS)—catalyzes conversion of L-arginine to NO in an oxygen- and NADPH-dependent reaction requiring cofactors such as tetrahydrobiopterin (BH_4_) [3,15]. These isoforms exert distinct physiological and pathological roles. eNOS-derived NO primarily maintains vascular homeostasis, regulating vascular tone, platelet activity, and endothelial function in cardiovascular and renal tissues [16]. nNOS mainly participates in neuronal signaling and local tissue regulation, including tubuloglomerular feedback and organ-specific microcirculatory control [17,18]. In contrast, iNOS is typically induced during inflammatory conditions and generates high levels of NO, which may contribute to nitrosative stress and tissue injury when dysregulated [19].

Because these pathways have been extensively characterized, subsequent sections emphasize how developmental stage, redox balance, and environmental exposures modulate NOS activity and the balance among NOS isoforms across the life course.

The nitrate–nitrite–NO axis represents an important complementary pathway, providing an alternative and diet-modifiable source of NO, particularly under hypoxic or acidic conditions [20,21]. Dietary nitrate is reduced to nitrite by oral microbiota and subsequently converted to NO systemically, especially when NOS activity is limited [22]. Human skin also contributes to NOS-independent NO production, as commensal bacteria reduce nitrate to nitrite, generating a local NO source [20]. Proteins such as deoxyhemoglobin and xanthine oxidoreductase further catalyze nitrite reduction under low oxygen tension. This nitrate-derived NO is particularly relevant during early development, in childhood, and in states of endothelial dysfunction, serving as an adaptive reserve system and representing a readily modifiable route for cardiovascular and renal protection.

NO synthesis is tightly regulated by substrate availability, endogenous inhibitors, redox state, and post-translational modifications [23,24]. The “arginine paradox” reflects compartmentalized substrate delivery and competitive inhibition [25,26]. BH_4_ deficiency leads to NOS uncoupling, shifting electron flow toward superoxide generation and amplifying oxidative stress [27]. Endogenous methylarginines such as ADMA and SDMA further limit NO bioavailability and are strongly associated with cardiovascular and renal disease [28]. Oxidative stress, inflammation, and aging disrupt NOS signaling via BH_4_ oxidation and impaired mechanotransduction [5,29]. External modifiers, including antiseptic mouthwash and proton pump inhibitors, may impair the nitrate–nitrite–NO pathway [30]. Collectively, NO production is dynamically regulated by developmental context, systemic redox environment, and contributions from both NOS-dependent and nitrate-reductive pathways—features central to life-course vulnerability.

### 3.2. NO Signaling in Cardiovascular–Kidney–Metabolic Health

#### 3.2.1. Downstream Signaling Mechanisms

NO signals primarily through activation of soluble guanylate cyclase (sGC), increasing cyclic GMP (cGMP) and activating protein kinase G (PKG), which reduces intracellular Ca^2+^ and induces smooth muscle relaxation [31,32]. Beyond vascular tone, this pathway modulates endothelial integrity and renal function [32,33].

NO also mediates cGMP-independent signaling through S-nitrosylation, which involves the transfer of an NO^+^ moiety (nitrosating equivalent) to cysteine residues, thereby distinguishing it from other redox-based NO signaling mechanisms, as well as through metal coordination reactions [34]. Mitochondrial targets, including cytochrome c oxidase, link NO availability to respiratory control and redox balance. Reaction with superoxide forms peroxynitrite, generating nitrated biomolecules and electrophilic lipid mediators with context-dependent biological effects [2,35,36]. Importantly, peroxynitrite can also inhibit sGC, thereby attenuating cGMP-dependent signaling and linking oxidative/nitrative stress to dysregulated NO downstream actions. Signal termination occurs via phosphodiesterases and rapid scavenging by hemoproteins such as hemoglobin [37,38].

**Figure 1:**
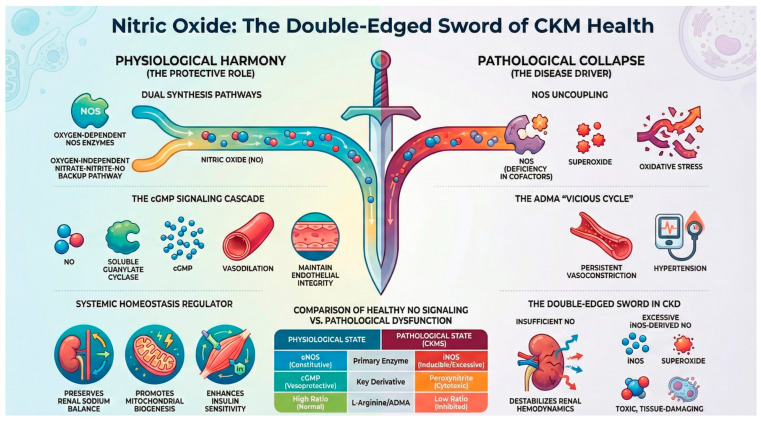
Context-dependent and life-course-modulated roles of nitric oxide in cardiovascular–kidney–metabolic health and disease.

The specificity of NO signaling derives from subcellular compartmentalization, concentration gradients, and redox coupling, rather than simplistic binary classifications. As illustrated in Figure 1, physiological NO signaling supports vascular, renal, and metabolic homeostasis, whereas impaired bioavailability or redox imbalance shifts signaling toward oxidative/nitrative stress and progressive CKMS pathology.

#### 3.2.2. NO in Cardiovascular–Kidney–Metabolic (CKM) Health Across the Life Course

NO functions as a central integrator of cardiovascular, renal, and metabolic homeostasis, with effects tightly constrained by its short half-life and local redox milieu [39].

In the cardiovascular system, endothelial NO maintains vascular compliance, suppresses platelet aggregation, limits leukocyte adhesion, and restrains vascular smooth muscle proliferation [8,16,17,32]. In the kidney, NO modulates afferent arteriolar tone, tubuloglomerular feedback, and tubular sodium handling, supporting pressure natriuresis and long-term blood pressure regulation [9,17,18,33].

Importantly, during nephrogenesis, NO signaling influences ureteric bud branching, nephron endowment, and intrarenal vascular development [40,41]. Early-life impairment—often reflected by elevated ADMA or reduced arginine-to-ADMA ratio—establishes structural and epigenetic trajectories predisposing to salt sensitivity, hypertension, and CKMS progression.

Metabolically, NO modulates mitochondrial respiration, oxidative phosphorylation efficiency, and reactive oxygen species balance [42,43]. It enhances insulin sensitivity and coordinates with AMP-activated protein kinase (AMPK) to integrate endothelial and metabolic signaling [44,45]. Through both NOS-dependent and nitrate-reductive pathways, the NO–mitochondria–AMPK axis mechanistically intertwines vascular and metabolic health across developmental stages.

## 4. The Pathological Face of NO in CKMS

The triad of CKMS is often driven by NO deficiency and oxidative stress. Pathological conditions, such as hyperglycemia and hypertension, lead to the accumulation of ADMA, an endogenous NOS inhibitor. This results in eNOS uncoupling, where the enzyme produces superoxide radicals instead of NO, further fueling a cycle of nitro-oxidative stress and endothelial dysfunction [2].

### 4.1. Obesity

Obesity represents a central etiological driver of CKMS, primarily through the convergence of chronic low-grade inflammation, dysregulated lipid metabolism, and neurohormonal activation [12,13]. Excess adiposity amplifies RAS activity and promotes systemic insulin resistance, together precipitating endothelial dysfunction and vascular stiffening that destabilize CKM homeostasis [46].

At a molecular level, the progression toward obesity is closely associated with a collapse of NO bioactivity, characterized by a pathological triad of reduced NO availability, accumulation of ADMA, and enhanced formation of peroxynitrite [10]. Under physiological conditions, NO supports metabolic flexibility by sustaining mitochondrial efficiency and promoting brown adipose tissue thermogenesis via upregulation of uncoupling protein 1 (UCP1) [47]. When NO signaling is impaired, this thermogenic capacity diminishes, leading to lower energy expenditure and increased feed efficiency [47].

In obese states, dysfunctional adipose tissue releases inflammatory mediators and metabolites such as kynurenine, which elevate ADMA levels and upregulate arginase activity, further constraining L-arginine availability for NO synthesis [48,49]. Rising ADMA promotes eNOS uncoupling, shifting the arginine/ADMA balance toward a pro-oxidant milieu and accelerating peroxynitrite generation [2]. This cascade reinforces insulin resistance, mitochondrial dysfunction, and progressive adiposity.

In summary, the transition to an obese phenotype can be conceptualized as a failure of the NO pathway to preserve mitochondrial integrity and adaptive thermogenesis; sustained ADMA elevation and nitrosative stress undermine core energy-regulatory mechanisms, thereby entrenching the CKMS trajectory.

### 4.2. Cardiovascular Disease

CVD is frequently initiated and exacerbated by a triad of factors: NO deficiency, elevated ADMA, and the formation of peroxynitrite. Endothelial dysfunction, mainly characterized by a reduction in NO bioavailability, is the initial event in the development of CVD [50]. Without sufficient NO to activate sGC, levels of cGMP fall, leading to impaired vasodilation and persistent vasoconstriction. This loss of NO bioactivity also fosters a pro-inflammatory environment, increasing the expression of adhesion molecules that recruit leukocytes to the endothelium, thereby triggering atherogenesis [50]. Furthermore, reduced NO availability in macrovessels promotes vessel wall remodeling and vessel constriction.

The presence of superoxide anions leads to a rapid reaction with any residual NO to form peroxynitrite (ONOO^−^), a potent and highly reactive oxidant [2]. Peroxynitrite induces nitrative stress, which causes single-strand DNA breaks and the nitration of tyrosine residues in proteins, such as 3-nitrotyrosine, which is commonly found in atherosclerotic plaques [51]. Peroxynitrite further degrades the vascular environment by inactivating critical enzymes like prostacyclin synthase and sGC, while also oxidizing the essential cofactor tetrahydrobiopterin (BH4) to BH2. The depletion of BH4 creates a “vicious cycle” where eNOS remains uncoupled, continuously generating superoxide and peroxynitrite, which further scavenges available NO behind endothelial dysfunction [52].

Clinically, high circulating ADMA and the subsequent nitro-oxidative stress are independent risk factors for cardiovascular morbidity and mortality [53]. This interplay between enzymatic inhibition and oxidative damage promotes myocardial remodeling, increased arterial stiffness, and severe endothelial dysfunction, ultimately leading to myocardial infarction or stroke. In cardiac tissues, uncoupled NOS activity mediates diastolic dysfunction and aggravates pressure-overload-induced hypertrophic remodeling. In conclusion, the transition from healthy vascular signaling to cardiovascular disease is driven by the collapse of the NO system under the combined weight of ADMA-mediated inhibition and peroxynitrite-induced nitrative damage.

### 4.3. Chronic Kidney Disease

CKD is increasingly recognized as a state in which the NO system functions as a quintessential “double-edged sword”. The biological impact of NO in the kidney is fundamentally context-dependent—shaped by its concentration, enzymatic source, spatial localization, and redox milieu [54,55]. Whereas physiological NO signaling is indispensable for renal autoregulation and vascular homeostasis, deviations in either direction—insufficient NO bioavailability or pathological NO excess—actively drive ongoing renal injury through distinct but converging mechanisms that sustain CKD progression.

On the side of NO deficiency, impaired constitutive NO production by eNOS and nNOS commonly arises from NOS uncoupling. Depletion of L-arginine or BH4, or competitive inhibition by ADMA, diverts NOS from NO synthesis toward superoxide generation, converting a protective enzyme into a pro-oxidant source [55]. This dual hit—reduced NO and increased oxidative stress—compromises endothelial function and destabilizes renal autoregulatory mechanisms, particularly the myogenic response and TGF, leading to unstable glomerular pressure dynamics, intraglomerular hypertension, and heightened vulnerability to ischemic and shear stress-mediated injury in established CKD [55,56]. In this setting, low NO represents the “insufficient signaling” edge of the sword, perpetuating hemodynamic instability and microvascular dysfunction.

At the opposite extreme, excessive NO production—predominantly mediated by inducible NOS (iNOS)—constitutes the “toxic signaling” edge of the NO double-edged sword in diseased kidneys [57]. Unlike eNOS and nNOS, iNOS is calcium-independent and capable of generating sustained, high-magnitude NO fluxes that can exceed constitutive levels by up to three orders of magnitude [54]. In inflammatory or injurious CKD states, this NO surge reacts rapidly with superoxide to form peroxynitrite, amplifying nitro-oxidative stress and propagating renal damage through multiple pathways [58]. Peroxynitrite drives renal cellular injury by inducing lipid peroxidation, DNA damage, and protein tyrosine nitration while simultaneously impairing mitochondrial respiration through inhibition of cytochrome c oxidase and iron–sulfur enzymes [59], leading to bioenergetic failure in tubular epithelial cells. Within the glomerulus, excessive NO and peroxynitrite contribute to podocyte hypertrophy, cytoskeletal disorganization, foot process effacement, and eventual podocyte detachment, undermining the integrity of the filtration barrier and accelerating proteinuria and progressive renal decline [60].

Taken together, NO in established CKD embodies a true biological double-edged sword: insufficient NO bioavailability destabilizes renal hemodynamics and exacerbates microvascular dysfunction, whereas excessive iNOS-driven NO triggers a cascade of nitro-oxidative injury, mitochondrial dysfunction, and structural deterioration that hastens disease progression. Accordingly, maintaining an optimal NO redox balance—rather than simply increasing or suppressing NO—should remain the central therapeutic principle in CKD management [9].

### 4.4. Diabetes, Dyslipidemia, and MAFLD

NO serves as a master molecular regulator of metabolic homeostasis, but its biological impact is strictly concentration-dependent. Research indicates that both a deficiency in constitutive NO and a pathological excess of inducible NO function as primary drivers of insulin resistance, dyslipidemia, and metabolic-associated fatty liver disease (MAFLD) [61,62].

Under physiological conditions, basal levels of NO produced by eNOS are essential for maintaining mitochondrial biogenesis and energy expenditure through the activation of peroxisome proliferator-activated receptor gamma co-activator-1α (PGC1-α), a transcription co-activator that plays a key role in regulating mitochondrial biogenesis and energy metabolism in multiple tissues [63]. A state of NO deficiency—often triggered by eNOS uncoupling—leads to overproduction of glucose and fatty acids in liver further stimulates the secretion of insulin by the pancreatic B cells, and elicits further peripheral insulin resistance thereby establishing a vicious circle during NAFLD development and progression [64].

Furthermore, the accumulation of ADMA tonically suppresses NO synthesis and fuels a cycle of oxidative stress are related to the hallmark features of metabolic syndrome, including elevated cholesterol, impaired glucose tolerance, and NAFLD [65,66,67]. ADMA levels are tightly controlled by its degradation via dimethylarginine dimethylaminohydrolase (DDAH-1 and DDAH-2) and, to a lesser extent, by renal excretion [68]. As liver is a major organ with DDAHs for ADMA metabolism [69], thereby establishing a vicious circle during NAFLD progression.

Conversely, the pathological induction of iNOS produces sustained, high-output fluxes of NO in response to chronic low-grade inflammation [70]. While high NO is utilized for immune defense, its chronic overproduction in metabolic tissues is inherently cytotoxic. Excessive iNOS-derived NO reacts with superoxide radicals at a diffusion-controlled rate to form peroxynitrite, a highly reactive oxidant that contributes to insulin resistance, type 2 diabetes, and NAFLD [71]. High NO levels also directly interfere with the insulin-signaling cascade via S-nitrosylation, causing a profound loss of protein function and inhibiting downstream metabolic actions.

Metabolic health depends on a precise redox balance of NO. Low NO precipitates metabolic syndrome by disabling mitochondrial biogenesis and increasing oxidative stress, whereas high NO drives the syndrome through nitrosative damage to the insulin-signaling cascade. Consequently, restoring the L-arginine/ADMA ratio and mitigating peroxynitrite formation are essential therapeutic goals for managing metabolic disorders [10,72].

## 5. NO Across the Life Span: A Life-Course Perspective

**Figure 2:**
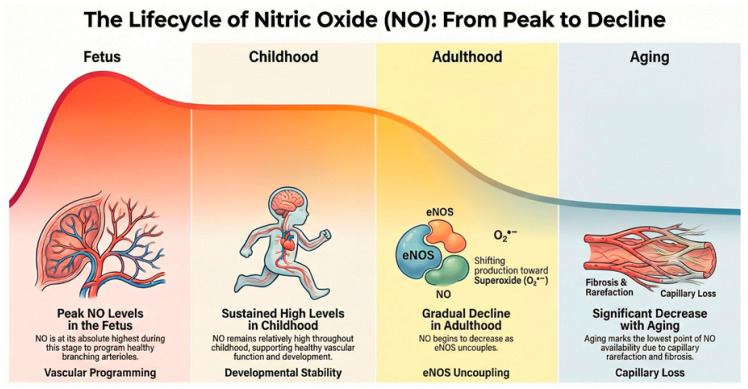
The nitric oxide lifespan: from fetal programming to aging resilience.

NO, recognized as “Molecule of the Year” in 1992 by *Science* [73], is a pleiotropic gaseous signaling molecule that governs physiological homeostasis from conception to senescence (Figure 2). Its multifaceted roles in vasodilation, neurotransmission, and immunity are concentration-dependent and highly sensitive to aging-related cellular changes. A life-course perspective reveals that the NOS/NO system acts as a determinant of human longevity, with its dysregulation serving as the common soil for CKMS.

### 5.1. Fetal and Early-Life Programming

During pregnancy, NO plays a central role in fetal and early-life programming by coordinating implantation, placental vascularity, embryonic development, placental angiogenesis, and overall fetal development [74]. Produced by constitutive NOS, NO modulates feto-placental blood flow, ensuring an adequate nutrient supply to the developing fetus.

In pregnant mice, all three NOS isoforms are expressed in uterine tissue from gestational days 4 to 8, underscoring the involvement of NO in implantation mechanisms [75]. In humans, circulating nitrate/nitrite levels are elevated in normal pregnancy [76], whereas impaired NO bioavailability and increased ADMA are associated with adverse pregnancy outcomes [77]. In early gestation, low ADMA with concomitantly high NO facilitates uterine relaxation and hemodynamic adaptation, thereby supporting normal fetal growth. In contrast, the physiological rise in ADMA during late pregnancy counterbalances NO-mediated relaxation to enhance uterine contractility and enable successful parturition [76,77].

Maternal plasma L-arginine is reduced in pregnancies complicated by intrauterine growth restriction (IUGR) [78], and both L-arginine levels and placental eNOS expression are lower in preeclampsia compared with healthy controls [79]. Consistently, both NOS inhibitors, ADMA and SDMA, concentrations are elevated in preeclamptic pregnancies [80,81]. Experimental models further demonstrate decreased L-arginine, reduced NO production, and enhanced superoxide generation, leading to excessive peroxynitrite formation in preeclamptic placentas [82]. Collectively, these findings indicate that precise, temporally regulated balance within the NOS/NO–ADMA axis is critical for placental function, fetal development, and the programming of later-life health.

Preclinical models demonstrate that impaired NOS/NO signaling (via L-NAME or ADMA) disrupts branching morphogenesis [40,83], reduces nephron number, and alters the renal transcriptome and key pathways (e.g., renin–angiotensin–aldosterone system) [41,84,85], supporting a mechanistic link between NO deficiency and kidney programming. In parallel, NO is an essential downstream mediator of VEGF-driven angiogenesis, and its deficiency constrains placental vascular development, further compounding IUGR and cardiovascular programming [8]. Metabolic programming is critically regulated by NO-dependent pathways that govern energy expenditure, mitochondrial function, and adipocyte differentiation [86]. The eNOS–cGMP–PGC1-α axis constitutes a master regulator of mitochondrial biogenesis and respiratory efficiency [63]; consistent with this, eNOS-deficient mice develop a full metabolic syndrome phenotype—obesity, dyslipidemia, and hypertension—even under normal caloric intake, underscoring the indispensability of constitutive NO bioactivity for metabolic homeostasis [87].

### 5.2. Childhood and Adolescence: Silent Trajectory Shaping

During childhood and adolescence, the NO system undergoes a distinct biochemical transition, with circulating ADMA displaying a U-shaped relationship with age. Neonatal ADMA levels are markedly high (~1.06 µM/L in cord blood) and decline at an average rate of 15 nM per year until approximately 25 years of age, paralleling the rapid maturation of kidney function in early life [88,89]. This period constitutes a “silent trajectory” during which subclinical vascular changes begin, with endothelial dysfunction—manifested as impaired NO production—preceding structural vascular alterations such as arterial stiffness by decades.

In children with early CKD, SDMA is a more sensitive marker of declining glomerular filtration rate (GFR) than creatinine, reflecting early impairment of renal homeostasis [90]. Preserving NO bioavailability during these formative years is critical for maintaining neurovascular coupling, supporting cognitive development, and preventing future metabolic syndrome [91]. Childhood obesity represents a major global health burden, affecting approximately 160 million children and adolescents aged 5–19 years in 2022, with projections suggesting that over half of the world’s population may be overweight or obese by 2035 [92,93]. Importantly, pediatric obesity serves as a primary driver of Stage 1 CKMS, characterized by metabolic dysregulation without overt structural kidney or cardiovascular damage, and is closely linked to perturbations in the NO/ADMA system [94,95,96].

### 5.3. Adulthood: Amplification of NO Dysregulation

In adulthood, cumulative exposure to Western diets—high in sugar, fat, and salt—physical inactivity, and environmental pollutants profoundly amplifies NO dysregulation [97,98,99]. This stage is marked by the clinical emergence of the CKM triad, including obesity, diabetes, and hypertension, all rooted in a common molecular substrate of NO dysregulation. Increasing evidence indicates that NO signaling is tightly intertwined with oxidative stress and inflammatory pathways, forming a self-reinforcing cycle that accelerates cardiometabolic and renal disease progression [70].

A primary mechanism is eNOS uncoupling, wherein chronic hyperglycemia and elevated free fatty acids generate excessive ROS that oxidize BH4 to BH2. In this state, eNOS produces superoxide rather than NO, facilitating the interaction of NO with superoxide to form the reactive nitrogen species peroxynitrite and leading to nitrative stress and damage to vascular proteins, DNA, and lipids [2]. Peroxynitrite and other RNS further impair endothelial function by oxidizing critical cofactors and amplifying redox imbalance, thereby propagating oxidative and inflammatory signaling. Concurrently, the systemic accumulation of ADMA and SDMA competitively inhibits L-arginine binding, reinforcing eNOS uncoupling and endothelial dysfunction. Elevated ADMA levels are clinically predictive of coronary artery disease, CKD progression, and increased cardiovascular mortality [53].

Oxidative stress also interacts closely with inflammatory pathways. Pro-inflammatory cytokines and immune cell activation enhance ROS production and further reduce NO bioavailability, while diminished NO signaling weakens its normal anti-inflammatory and vasoprotective effects on the endothelium [70]. NO deficiency further disrupts metabolic homeostasis by impairing mitochondrial biogenesis via the eNOS–cGMP–PGC1-α pathway [63]. Reduced NO bioavailability in obese or sedentary adults decreases brown adipose tissue thermogenesis and increases feed efficiency, favoring fat storage. Moreover, chronic low-grade inflammation commonly observed in metabolic disorders induces iNOS, producing micromolar NO fluxes that readily react with ROS and promote nitrosative stress. This process can S-nitrosylate insulin signaling proteins such as IRS-1 and Akt, contributing to insulin resistance [72].

Environmental pollutants—including air pollution and industrial toxins like 2,3,7,8-tetrachlorodibenzo-p-dioxin (TCDD)—exacerbate these effects by promoting inflammation, mitochondrial destabilization, and an imbalance in the NO:superoxide ratio, accelerating vascular stiffening and arterial remodeling [100]. Collectively, adulthood represents a critical window where lifestyle and environmental exposures converge to collapse NO signaling, through the combined actions of oxidative stress, reactive nitrogen species formation, and inflammation, transforming a previously healthy vascular and metabolic state into a diseased CKM phenotype. Restoration of the L-arginine:ADMA balance and enhancement of NO bioavailability remain key therapeutic strategies.

### 5.4. Aging: Decline in NO Resilience

As humans enter the geriatric phase, the NO system undergoes a marked decline in resilience, contributing to systemic “inflammaging” [101]. Multiple animal models indicate that intestinal NO homeostasis and arginine metabolism mediated through arginase and NO synthesis is altered in small intestine of aging mice [102]. Plasma ADMA levels, which decline during early adulthood, rise again in older age, reaching concentrations nearly twice those of young adults, coinciding with a reduction in endothelium-derived NO [103]. This loss undermines the antioxidant, vasodilatory, and metabolic functions of NO across multiple organ systems.

Several convergent mechanisms drive this decline. Chronic oxidative stress irreversibly oxidizes BH4 to BH2, trapping eNOS in an uncoupled state that generates superoxide instead of NO [104]. Age-associated loss of SIRT1 activity promotes eNOS hyperacetylation, suppressing enzymatic function and accelerating endothelial senescence [105]. Additionally, NO-stimulated mitochondrial biogenesis via PGC1-α diminishes, resulting in mitochondrial fragmentation, bioenergetic deficits, and reduced adaptive capacity in cardiac and metabolic organs [106]. The collapse of NO resilience directly contributes to age-related cardiovascular decline. In the kidney, senescence of the NO system promotes peritubular capillary rarefaction, tubulointerstitial injury, and susceptibility to CKD [107]. Collectively, these alterations highlight how the interplay of oxidative stress, ADMA accumulation, and enzymatic/epigenetic dysfunction transforms aging into a systemic NO-deficient state, underpinning multi-organ vulnerability and CKMS risk.

Maintaining NO homeostasis throughout life is therefore critical for longevity. While fetal and early-life stages establish the NO-dependent foundation of organ systems, aging tests this resilience. Early-life reprogramming strategies, combined with precision NO-targeted interventions in adulthood, represent a promising approach to mitigate the cumulative burden of CKMS.

To counteract the progressive life-course dysregulation of NO, emerging therapeutic strategies increasingly center on restoring and sustaining NO bioactivity. These approaches encompass both early prevention—designed to harness developmental plasticity to optimize NO signaling and avert the initiation of CKMS—and NO-targeted therapies aimed at slowing or modifying disease progression once CKMS is established.

## 6. Early Prevention via NO Trajectory Reprogramming

Early prevention seeks to exploit critical windows of developmental plasticity to reestablish NO bioavailability and thereby reduce the likelihood of early CKMS onset. This reprogramming strategy is fundamentally anchored in the restoration of the NO pathway, a central regulator of vascular tone, nephrogenesis, and cellular redox balance.

Multiple mechanistic avenues exist to enhance NO bioavailability, including supplementation of NOS substrates (e.g., L-arginine or L-citrulline), reduction in endogenous NOS inhibition via ADMA modulation, administration of NO donors, and direct or indirect enhancement of NOS activity.

Table 1 summarizes representative experimental studies in which NO-based reprogramming interventions were implemented prior to CKMS onset, demonstrating their potential to durably modify cardiometabolic and renal trajectories across the life course [40,108,109,110,111,112,113,114,115,116,117,118,119,120,121,122,123,124,125,126,127].

A variety of NO-targeted strategies have been explored to enhance NO bioavailability. To date, most of these approaches have been evaluated for their potential to perinatally reprogram cardiovascular and kidney development, whereas their relevance to metabolic programming remains largely unexplored. Each of these strategies is discussed in turn.

### 6.1. NOS Substrates

L-arginine supplementation has been widely used to enhance NO production in experimental settings [128,129]. However, in most studies, L-arginine was not administered during critical windows of pregnancy and lactation, thereby limiting insight into its potential role in developmental reprogramming. To date, only two studies have demonstrated that maternal supplementation with L-arginine combined with antioxidants can protect fawn-hooded hypertensive rats against hypertension, proteinuria, and glomerulosclerosis in adulthood [108], and prevent elevated blood pressure in spontaneously hypertensive rats (SHRs) [109]. Notably, L-arginine is not an optimal NO precursor, as it is involved in multiple competing metabolic pathways.

L-citrulline, the primary precursor of L-arginine, serves as an alternative substrate for NO synthesis [130]. Compared with L-arginine, L-citrulline exhibits superior bioavailability because it bypasses hepatic first-pass metabolism. L-citrulline is converted back to L-arginine primarily in the kidney, although extra-renal tissues also contribute to this recycling pathway [131]. Accumulating evidence from animal models indicates that maternal L-citrulline supplementation effectively protects adult offspring from hypertension of developmental origin, including models of streptozotocin-induced maternal diabetes [48], maternal caloric re-striction [110], prenatal dexamethasone exposure [111], maternal N^G^-nitro-l-arginine methyl ester (L-NAME) administration [112], and maternal adenine-induced CKD [113]. In SHRs, maternal L-citrulline supplementation enhanced renal NO bioavailability and prevented the development of hypertension [114]. Moreover, early-life L-citrulline treatment in young SHRs has been shown to halt the progression from prehypertension to established hypertension [115].

Although L-citrulline supplementation has also been reported to attenuate hepatic lipid accumulation and prevent hypertriglyceridemia in adult rats exposed to a high-fructose diet [132], whether maternal supplementation with NOS substrates can restore NO signaling and thereby prevent metabolic programming remains largely unexplored and warrants further investigation.

### 6.2. ADMA-Lowering Agents

Another strategy to enhance NO bioavailability involves lowering ADMA, an endogenous NOS inhibitor. Although a specific ADMA-lowering agent is not yet available for clinical use, several widely prescribed drugs and bioactive compounds have been shown to restore NO availability through modulation of ADMA metabolism.

By augmenting the expression and/or activity of ADMA-metabolizing enzymes, particularly DDAHs, multiple interventions have been demonstrated to lower ADMA levels and reprogram cardiovascular and renal development, thereby preventing offspring hypertension. These include resveratrol, garlic oil, N-acetylcysteine, and melatonin in models of prenatal dexamethasone plus TCDD exposure [116], maternal CKD [117], prenatal dexamethasone combined with a postnatal high-fat diet [118], and maternal high-fructose plus postnatal high-salt diets [129], respectively. In addition, maternal treatment with the Nrf2 activator dimethyl fumarate prevented two-hit-induced programmed hypertension in male offspring, an effect associated with reduced ADMA levels [120].

Beyond maternal interventions, early postnatal treatment with aliskiren, a renin inhibitor approved for hypertension management, has been shown to prevent adult hypertension accompanied by ADMA reduction in offspring born to calorie-restricted dams when administered between 2 and 4 weeks of age [121]. Moreover, maternal supplementation with lactoferrin, a glycoprotein derived from breast milk, prevented offspring hypertension induced by maternal CKD, an effect linked to decreased ADMA levels [122]. Whether these interventions predominantly inhibit ADMA-producing enzyme protein arginine methyltransferase (PRMT) or enhance ADMA-metabolizing pathways requires further clarification.

Several commonly prescribed drugs recommended for the management of CKMS have also demonstrated ADMA-lowering effects in both human and animal studies. Agents such as telmisartan [133], glucagon-like peptide-1 receptor agonists [134], and rosuvastatin [135] reduce ADMA levels by suppressing ADMA-generating enzymes, whereas metformin [136], atorvastatin [137], and rosuvastatin [138] enhance the activity or expression of ADMA-metabolizing enzymes. Notably, metformin has additionally shown protective effects against liver steatosis in offspring exposed to a maternal high-fat diet [139]; however, whether its developmental reprogramming effects are directly mediated through ADMA lowering remains uncertain.

Despite accumulating experimental evidence, the lack of specific ADMA-lowering agents limits current clinical translation. The development of targeted PRMT inhibitors and DDAH agonists may offer future therapeutic opportunities to reduce ADMA levels [140,141], restore NO bioavailability, and prevent adverse cardiovascular–kidney–metabolic programming.

### 6.3. NO Donors

NO donors comprise a heterogeneous group of compounds capable of releasing NO through enzymatic or non-enzymatic mechanisms, independent of endogenous NOS activity [142,143]. These agents are broadly categorized into direct donors, which spontaneously liberate NO (e.g., sodium nitrite), and indirect donors that require metabolic or enzymatic activation. Among them, N-diazeniumdiolates (NONOates) are distinguished by their predictable release kinetics, generating two molar equivalents of NO upon spontaneous hydrolysis. S-nitrosothiols, such as S-nitrosoglutathione (GSNO), function as endogenous NO reservoirs and mediators of protein S-nitrosylation, exhibiting comparatively lower cytotoxicity. Advances in nanotechnology and targeted delivery systems have been explored to overcome the extremely short half-life and high reactivity of NO.

Despite substantial progress in NO donor development [142,143], their potential role in developmental or early-life reprogramming remains poorly defined. Classical nitrodilators, including nitroglycerin, pentaerythritol tetranitrate (PETN), and molsidomine, exert NO-mimetic vasodilatory effects via exogenous NO release [144,145]. Experimental studies demonstrate that perinatal exposure to PETN or molsidomine attenuates offspring hypertension in SHRs and FHH rats, respectively [102,103]. Similarly, early-life sodium nitrate supplementation prevents the transition from prehypertension to established hypertension in young SHRs [115]. Consistent with this concept, our recent work showed that early administration of the NO donor diethylenetriamine/NO adduct (DETA NONOate) effectively blunted BP elevation in young rats with CKD [125], supporting a time-dependent window for NO-based interventions.

While studies in animal models suggest that NO donors during the perinatal period or early postnatal life can modulate developmental pathways, translation to human infants is limited by species differences in NO metabolism, dosing, and developmental timing. Moreover, the safety profile of NO donors in this population remains incompletely characterized, warranting caution and further investigation before clinical application.

### 6.4. Other Strategies

Emerging evidence suggests that several nonconventional interventions may enhance NO signaling during early life. Melinjo (Gnetum gnemon) seed extract supplementation during lactation has been shown to upregulate eNOS expression and prevent maternal high-fructose-diet-induced hypertension in adult female offspring [126]. Similarly, maternal melatonin treatment improves myocardial resilience in offspring exposed to prenatal hypoxia, an effect associated with increased cardiac eNOS protein abundance [127].

Beyond increasing NOS expression, alternative strategies may improve NO bioavailability by enhancing NOS activity. Pharmacologic agents targeting the renin–angiotensin-aldosterone system (RAAS) or statins have been reported to restore NOS function by attenuating oxidative stress and preserving NO signaling [28]. In addition, modulation of post-translational NOS modifications—such as phosphorylation, S-glutathionylation, or coupling status—represents a promising yet underexplored approach for optimizing NO bioavailability in early-life prevention strategies.

## 7. Drugs for CKMS and Their Interaction with NO

Pharmacologic management of CKMS is mechanism-driven and stage-adapted, aiming not only to slow kidney disease progression and reduce cardiovascular risk but also to restore NO bioavailability. Endothelial dysfunction and impaired NO signaling—driven by oxidative stress, inflammation, RAAS overactivation, and metabolic dysregulation—represent a unifying pathophysiologic axis in CKMS. This section summarizes the major evidence-based drug classes used in CKMS and delineates their mechanistic interactions with NO pathways.

### 7.1. RAAS Blockade

The RAAS and the NO system exist in a state of functional antagonism [146]. Chronic RAAS overactivation, particularly via angiotensin II (Ang II), promotes NO deficiency by stimulating NADPH oxidase-derived superoxide generation, which rapidly scavenges NO to form peroxynitrite [147]. Ang II also suppresses DDAH activity, leading to ADMA accumulation and subsequent eNOS uncoupling [148]. In obesity, activation of the classical RAAS axis enhances lipogenesis, inhibits lipolysis, and drives adipocyte growth and differentiation, whereas activation of the non-classical RAAS axis improves lipid metabolism and insulin sensitivity while attenuating inflammation and obesity [149,150].

Angiotensin-converting enzyme (ACE) inhibitors (e.g., enalapril) and angiotensin II receptor blockers (ARBs, e.g., losartan, valsartan) constitute the guideline-recommended cornerstone of CKMS therapy [13]. Beyond BP and proteinuria reduction, RAAS blockade improves endothelial function by mitigating Ang II-induced oxidative stress and preserving NO bioavailability [151]. ACE inhibitors further enhance bradykinin-mediated eNOS activation, promoting vasodilation and cardiovascular protection [152], while certain ARBs, such as telmisartan, have demonstrated ADMA-lowering effects [153].

### 7.2. Glucagon-like Peptide-1 Receptor Agonists (GLP-1 RA)

Glucagon-like peptide-1 receptor agonists (GLP-1RAs) have gained increasing attention for their potential benefits in people with type 2 diabetes with CKD. Accumulative evidence indicates that GLP-1RAs can reduce albuminuria, mitigate cardiovascular risk, and attenuate kidney function decline [153]. Liraglutide, semaglutide, and dulaglutide provide integrated metabolic and cardiovascular benefits and are now foundational disease-modifying agents in CKMS [13].

GLP-1 receptor agonists can reduce ADMA levels through oxidative stress reduction, DDAH preservation [134]. Additionally, GLP-1 receptor activation enhances eNOS phosphorylation via AMPK and PI3K–Akt pathways, providing a mechanistically credible link between GLP-1 therapy and improved endothelial function [154]. These effects contribute to improved endothelial function, reduced arterial stiffness, and attenuation of atherosclerotic progression, alongside weight loss and glycemic control.

### 7.3. Sodium–Glucose Cotransporter-2 (SGLT2) Inhibitors

Sodium–glucose cotransporter-2 (SGLT2) inhibitors are foundational therapies for CKMS with substantial interaction with the NO system. Their cardiorenal protective effects are mediated in part by restoration of renal hemodynamic autoregulation through TGF, a process critically dependent on nNOS activity in the macula densa [155]. By increasing distal sodium delivery, SGLT2 inhibitors reactivate nNOS-dependent TGF, thereby reducing glomerular hyperfiltration and preventing pressure-induced kidney injury [156].

Beyond hemodynamics, metabolic programming is tightly regulated by NO-dependent pathways controlling energy expenditure and mitochondrial biogenesis via the eNOS–cGMP–PGC-1α axis [157]. SGLT2 inhibitors indirectly support this axis by alleviating systemic hyperglycemia and oxidative stress, preserving BH4 availability, and maintaining eNOS in a coupled, NO-producing state. Although ADMA is more directly targeted by RAAS blockade and metformin, SGLT2 inhibitors complement these strategies by stabilizing the renal and metabolic microenvironment [158]. This stabilization contributes to the preservation of systemic NO resilience [159], the progressive loss of which across the life course underlies cumulative cardiometabolic and multi-organ vulnerability.

### 7.4. Non-Steroidal Mineralocorticoid Receptor Antagonists (MRAs)

Non-steroidal mineralocorticoid receptor antagonists (MRAs), such as finerenone, are foundational therapies for CKMS, particularly in patients with CKD and persistent albuminuria [13,160]. The relationship between MRAs and NO is primarily mediated through the reduction in oxidative stress and the restoration of NO [161].

In CKMS, excessive aldosterone signaling triggers a pathological cascade involving the activation of the NLRP3 inflammasome and the overproduction of ROS from NADPH oxidase and mitochondria [162]. By selectively blocking the mineralocorticoid receptor, MRAs dampen this cycle of nitro-oxidative stress. Restored NO bioactivity then acts as an endogenous defense by suppressing inflammasome activation and mitochondrial bioenergetic failure. Furthermore, adequate NO signaling is vital for maintaining the glomerular filtration barrier by protecting the actin cytoskeleton of podocytes, thereby preventing the structural effacement and detachment associated with progressive proteinuria [163].

### 7.5. Lipid-Lowering Therapy

Hydroxy-methyl-glutaryl–coenzyme A (HMG-CoA) reductase inhibitors (statins) reduce atherosclerotic cardiovascular risk in CKMS [164]. Their primary lipid-lowering effect is mediated by inhibition of hepatic cholesterol biosynthesis, leading to upregulation of low-density lipoprotein (LDL) receptors and enhanced LDL-cholesterol clearance [164]. Beyond lipid reduction, statins exert pleiotropic actions, including suppression of proinflammatory cytokines, attenuation of ROS generation, and inhibition of epithelial–mesenchymal transition-related signaling pathways [165]. Importantly, statins enhance endothelial NO bioavailability by upregulating eNOS expression and activity while reducing oxidative stress and ADMA levels [137,138]. Collectively, these effects substantially contribute to the cardiovascular protection conferred by statins in CKD [166].

### 7.6. Adjunct and Emerging Therapies

Endothelin receptor antagonists and selective uric acid-lowering therapies may further influence NO pathways by reducing endothelial inflammation, oxidative stress, and metabolic load. These approaches are of particular interest as adjuncts aimed at correcting NO deficiency in advanced CKMS.

Endothelin-1 (ET-1) is a potent vasoconstrictor that acts as a direct antagonist to NO-mediated vasodilation [167]. In aging and sedentary states, enhanced ET-1 signaling drives eNOS uncoupling and tonic arterial constriction [168]. Endothelin receptor antagonists, such as macitentan, have been shown to improve endothelial dysfunction and vascular inflammation by protecting the NO pathway [169].

Selective uric acid-lowering therapies, such as xanthine oxidase (XO) inhibitors (e.g., febuxostat, allopurinol), have a complex, “double-edged” relationship with NO [170]. While XO is a major source of ROS that scavenge NO, it also functions as a critical nitrite reductase that generates NO under hypoxic or ischemic conditions. Consequently, while inhibiting XO can reduce oxidative stress, it may also abolish the “backup” nitrate–nitrite–NO pathway [171]. Chronic XO inhibition has been shown to worsen hypertension in eNOS-deficient models by removing this compensatory source of NO bioactivity [171]. Integration of these therapies therefore requires careful consideration to maintain optimal redox balance and NO homeostasis.

### 7.7. iNOS Inhibitors

As mentioned above, iNOS is a major source of high-output NO during inflammatory states in CKMS, driving nitro-oxidative stress through excessive NO and peroxynitrite formation. While this pathogenic role provides a strong mechanistic rationale for iNOS inhibition, clinical translation has remained elusive. Preclinical studies consistently demonstrate that suppressing iNOS attenuates insulin resistance, kidney injury, and cardiovascular remodeling [172,173,174]; however, these benefits have not translated into durable clinical efficacy [175].

A key barrier lies in the context-dependent duality of iNOS signaling [20]. Beyond its pathological induction, basal or compensatory iNOS activity contributes to vascular tone regulation, tissue perfusion, and metabolic homeostasis under stress conditions [19]. Broad or sustained iNOS inhibition therefore risks disrupting adaptive NO signaling, particularly in advanced disease states. Consistent with this complexity, multiple selective iNOS inhibitors have failed in clinical trials due to limited efficacy or unacceptable safety profiles.

Collectively, these findings suggest that the challenge of iNOS-targeted therapy in CKMS is not insufficient biological rationale, but rather the inability of current pharmacologic strategies to achieve tissue-, stage-, and context-specific modulation of iNOS activity [175].

### 7.8. Practical Therapeutic Hierarchy in Established CKMS

In clinical practice, RAAS blockade establishes the foundation for NO preservation and renal protection, followed by SGLT2 inhibitors as disease-modifying agents. GLP-1 receptor agonists are added in patients with obesity or type 2 diabetes to enhance metabolic and NO-mediated vascular benefits. Finerenone addresses residual inflammatory–fibrotic signaling and NO impairment in albuminuric CKD, while statins and individualized BP control provide complementary endothelial and cardiovascular protection.

Taken together, this therapeutic hierarchy reflects a shift from isolated risk-factor control toward integrated restoration of NO signaling across cardiovascular, renal, and metabolic domains. Rather than viewing CKMS as a static disease of late adulthood, this framework aligns pharmacologic decision-making with the cumulative life-course decline in NO bioavailability. In doing so, it positions established CKMS as the clinical manifestation of decades-long maladaptive NO signaling, and pharmacotherapy as a means of re-establishing physiological resilience rather than merely slowing end-stage progression.

## 8. Translational and Preventive Implications

Despite its central role in cardiovascular–kidney–metabolic regulation, therapeutic manipulation of NO remains intrinsically challenging. The extreme reactivity and short biological half-life of NO, coupled with rapid inactivation by hemoglobin and reactive oxygen species, severely constrain its spatial and temporal bioavailability [47], thereby limiting effective clinical delivery.

Traditional NO-based therapies, including inhaled NO and classical NO donors such as organic nitrates and sodium nitroprusside, have provided important clinical benefits but are fundamentally limited by systemic exposure, lack of tissue specificity, and the development of nitrate tolerance driven by oxidative stress and downstream signaling desensitization [176]. These limitations highlight a persistent translational gap between the biological importance of NO signaling and the clinical efficacy of NO-targeted interventions.

Recent advances in nanotechnology have introduced a conceptual shift in NO therapy—from transient systemic supplementation toward controlled, localized restoration of NO signaling [177]. Nanomaterial-based platforms enable stabilization of NO donors, modulation of release kinetics, and exploitation of endogenous NO reservoirs, thereby improving spatial precision and therapeutic durability [178]. Together, catalytic and noncatalytic strategies represent an emerging framework for context-dependent modulation of NO bioavailability rather than indiscriminate NO augmentation.

Building on this, we now explicitly recognize current knowledge gaps and future research directions. Specifically, studies are urgently needed to clarify stage-specific regulation of NO signaling across the life course, including how early-life perturbations in NO pathways contribute to long-term cardiovascular, renal, and metabolic risk.

Recognition of NO’s mechanistic duality has critical implications for CKMS management. Effective therapeutic strategies should aim not simply to increase or suppress NO levels, but to restore physiological NO signaling through preservation of eNOS coupling, attenuation of oxidative stress, and modulation of inflammatory pathways across different life stages. Preventive strategies targeting early-life NO homeostasis may yield disproportionate long-term benefits, highlighting opportunities for life-course risk modification and early reprogramming interventions.

This review extends the conceptual scope of the NO system beyond treatment of established CKMS to include developmental and age-specific reprogramming as innovative preventive strategies. Although numerous NO-related reprogramming interventions have demonstrated improved cardiovascular–kidney–metabolic outcomes in preclinical models, critical gaps remain regarding optimal dosage, timing, and duration of interventions across different life stages before clinical translation. Whether NO-based interventions during pregnancy, lactation, or early childhood confer additive benefits in preventing adult CKMS warrants evaluation in rigorously designed clinical trials involving populations at high risk for CKMS programming.

Collectively, these revisions emphasize not only therapeutic opportunities but also life-course-oriented preventive strategies, bridging the translational gap between NO biology and clinical application.

## 9. Conclusions

NO functions as a context-dependent, mechanistically bidirectional regulator in CKMS, acting as both a guardian of cardiovascular–kidney–metabolic health and a mediator of disease when dysregulated. Across the life span, NO signaling integrates developmental programming, environmental exposures, and aging-related decline. Recognition of this duality emphasizes the necessity of prevention-oriented, life-course strategies aimed at preserving NO homeostasis, offering a rational and biologically grounded pathway to reduce the global burden of CKMS.

## Figures and Tables

**Table 1:** NO-Based Reprogramming Strategies in CKMS Animal Models.

NO-Based Reprogramming Interventions	Timing	CKM Trajectories	Animal Model	Reprogramming Effects & Mechanisms	Ref.
NOS substrates					
L-arginine (20 g/L) in water + Antioxidants *	G/L	Kidney/cardiovascular	Genetic hypertension model, FHH	Prevented high BP, proteinuria, and glomerulosclerosis expression	[108]
L-arginine (20 g/L) in water + Antioxidants *	G/L	Cardiovascular	Genetic hypertension model, SHR	Prevented high BP	[109]
L-citrulline (250 g/L) in water	G/L	Kidney/cardiovascular	Streptozotocin-treated maternal diabetes	Prevented high BP and kidney damage and protected against reduced nephron number	[40]
L-citrulline (250 g/L) in water	G/L	Kidney	Maternal 50% caloric restriction	Prevented kidney damage and protected against reduced nephron number	[110]
L-citrulline (250 g/L) in water	G/L	Kidney/cardiovascular	Prenatal dexamethasone exposure	Prevented high BP	[111]
L-citrulline (250 g/L) in water	G/L	Kidney/cardiovascular	L-NAME-treated (Preeclampsia)	Prevented high BP and protected against reduced nephron number	[112]
L-citrulline (250 g/L) in water	G/L	Kidney/cardiovascular	Maternal adenine-induced CKD	Prevented high BP and kidney damage	[113]
L-citrulline (250 g/L) in water	G/L	Kidney/cardiovascular	Genetic hypertension model, SHR	Prevented high BP	[114]
L-citrulline (250 g/L) in water	C	Kidney/cardiovascular	Genetic hypertension model, SHR	Prevented high BP	[115]
ADMA-lowering agents					
Resveratrol (0.5 g/L) in water	G/L	Kidney/cardiovascular	Prenatal dexamethasone plus TCDD exposure	Prevented high BP	[116]
Garlic oil (100 mg/kg/day) via oral gavage	G/L	Kidney/cardiovascular	Maternal adenine-induced CKD	Prevented high BP	[117]
N-acetylcysteine (10 g/L) in water	G/L	Kidney/cardiovascular	Prenatal dexamethasone plus postnatal high-fat diet	Prevented high BP	[118]
Melatonin (0.1 g/L) in water	G/L	Kidney/cardiovascular	Maternal high-fructose plus postnatal high-salt diets	Prevented high BP	[119]
Dimethyl fumarate (50 mg/kg/day) via oral gavage	G	Kidney/cardiovascular	Prenatal dexamethasone plus postnatal high-fat diet	Prevented high BP	[120]
Aliskiren (10 mg/kg/day) via oral gavage	L	Kidney/cardiovascular	Maternal 50% caloric restriction	Prevented high BP	[121]
Lactoferrin (1 g/kg/day) in chow	G/L	Kidney/cardiovascular	Maternal adenine-induced CKD	Prevented high BP	[122]
NO donors					
Pentaerythritol tetranitrate (50 mg/kg/day) in chow	G/L	Cardiovascular	Genetic hypertension model, SHR	Prevented high BP	[123]
Molsidomine (120 mg/L) in water	G/L	Cardiovascular	Genetic hypertension model, FHH	Prevented high BP	[124]
Sodium nitrate (1 mmol/kg/d) in water	C	Kidney/cardiovascular	Genetic hypertension model, SHR	Prevented high BP	[115]
DETA NONOate (10 mg/kg/day) i.p.	C	Cardiovascular	Adenine-induced CKD	Prevented high BP	[125]
S-nitrosoglutathione (10 mg/kg/day) i.p.	C	Cardiovascular	Adenine-induced CKD	Prevented high BP	[125]
Enhancement of NOS					
Melinjo (Gnetum gnemon) seed extract 0.1% in chow	L	Cardiovascular	Maternal high-fructose diet	Prevented high BP	[126]
Melatonin (0.05 mg/kg/day) in water	G	Cardiovascular	Maternal hypoxia	Improves myocardial resilience	[127]

* Antioxidants: vitamins C and E, and taurine. G = gestation; L = lactation; C = Childhood; FHH = Fawn-hooded hypertensive rats; SHR = Spontaneously hypertensive rat; TCDD = 2,3,7,8-tetrachlorodibenzo-p-dioxin; i.p. = intraperitoneal; L-NAME = N^G^-nitro-l-arginine methyl ester; BP = blood pressure.

## Data Availability

No new data were created or analyzed in this study. Data sharing is not applicable to this article.
